# Culture and Metagenomic Insights into the Ear Microbiota in Dogs with Healthy Ears and Otitis Externa

**DOI:** 10.3390/vetsci13030250

**Published:** 2026-03-06

**Authors:** Emre Karakaya, İzzet Burçin Satıcıoğlu, Doğancan Yarım, Özgür Güran, Cansu Güran, Umut Alpman, Gültekin Atalan, Seçil Abay, Fuat Aydın

**Affiliations:** 1 Department of Microbiology, Faculty of Veterinary Medicine, Erciyes University, 38280 Kayseri, Türkiye; dogancanyarim35@gmail.com (D.Y.); sabay@erciyes.edu.tr (S.A.); faydin@erciyes.edu.tr (F.A.); 2 Department of Aquatic Animal Diseases, Faculty of Veterinary Medicine, Bursa Uludag University, 16059 Bursa, Türkiye; iburcinsat@gmail.com; 3 Institute of Aquaculture, Faculty of Natural Sciences, University of Stirling, Stirling FK9 4LA, UK; 4 Department of Veterinary Microbiology, Institute of Health Sciences, Erciyes University, 38039 Kayseri, Türkiye; ozgurguran@erciyes.edu.tr (Ö.G.); cansuguran@erciyes.edu.tr (C.G.); 5 Department of Surgery, Faculty of Veterinary Medicine, Erciyes University, 38280 Kayseri, Türkiye; umutalpman@erciyes.edu.tr (U.A.); gatalan@erciyes.edu.tr (G.A.)

**Keywords:** canine ear microbiota, otitis externa, culture, *16S rRNA* gene sequencing, shotgun metagenomic sequencing

## Abstract

The canine ear microbiota plays an important role in ear health, and dysbiosis is associated with otitis externa (OE) and antimicrobial resistance (AMR). This study aims to investigate the ear microbiota of dogs with healthy ears and OE using bacterial culture-based methods and shotgun metagenomic sequencing, and to screen for AMR and virulence-associated genetic signatures. Ear swab specimens from 100 healthy and 100 OE-affected dogs were analyzed. The isolates obtained via bacterial culture were identified by MALDI-TOF MS and *16S rRNA* sequencing. Metagenomic analysis was performed via Illumina shotgun sequencing. The most commonly defined species in healthy dogs in culture were *Staphylococcus pseudintermedius* (24.5%) and *Staphylococcus epidermidis* (5.7%); in dogs with OE, the most commonly defined species were *S. pseudintermedius* (30.5%), and *Clostridium perfringens* (4.5%). In healthy samples, metagenomic analysis revealed higher relative abundances of *Bacteroides fragilis* (15.8%) and *Ezakiella coagulans* (8.2%), while *S. pseudintermedius* (38.7%) dominated in OE. AMR profiling demonstrated diverse resistance determinants, including efflux pump systems and methicillin resistance-associated genes. In conclusion, the present study shows that *S. pseudintermedius* is a predominant member of canine ear microbiota, with higher presence in OE highlighting microbial shifts, and demonstrates that combining culture and metagenomic analyses provides a concise view of microbial communities and clinical relevance.

## Simple Summary

Microbiota refers to the living microorganisms present in a specific environment, such as the oral or gut microbiota, and in various parts of the body, including the oral cavity, intestines, skin, and ear canal. The ear canal microbiota of dogs with healthy and otitis externa (OE) poses a significant risk to human health due to the presence of important pathogenic microorganisms (*Staphylococcus* spp., *Pseudomonas aeruginosa*, and *Escherichia coli*). The present study aimed to investigate the ear microbiota of dogs with and without OE using bacterial culture-based techniques and shotgun metagenomic sequencing, and to assess antimicrobial resistance and virulence-associated genetic signatures through read-based analysis. Bacterial culture-based results highlight the polymicrobial nature of the canine ear canal, while metagenomic profiling reveals heterogeneous, pathogen-dominated communities in OE, predominantly characterized by *Staphylococcus pseudintermedius*.

## 1. Introduction

More people keep pets at home today, with dogs being the most popular. At least one in three households worldwide owns a dog [1]. Pets, especially dogs, contribute to their owners’ physical and mental health through a close bond with humans. However, as the frequency of keeping these animals in homes increases, so does the incidence of zoonotic and antimicrobial-resistant pathogenic microorganisms in humans [2,3]. The ear canal microbiota of dogs with healthy and otitis externa (OE) poses a significant risk to human health due to the presence of important pathogenic microorganisms (*Staphylococcus* spp., *Pseudomonas aeruginosa*, *Escherichia coli*, etc.) [4,5].

Otitis externa, defined as inflammation of the external ear canal, is a common disease in dogs and has a multifactorial etiology [6,7]. Factors that contribute to the occurrence of cases include heat, humidity, earwax buildup, age, gender, race, trauma, allergies, autoimmune diseases, endocrine disorders, anatomical structure, foreign bodies, ear parasites, bacterial infections, fungal infections, and other factors [5,6,8,9]. Microorganisms play a significant role in OE development, and the ear canal microbiota of dogs with OE harbors many pathogenic bacteria. Bacteriological analysis and next-generation sequencing (NGS)-based microbiome analyses reveal the presence of numerous Gram-positive bacteria (*Staphylococcus* spp., *Enterococcus* spp., and *Corynebacterium* spp.) as well as Gram-negative bacteria (*Pseudomonas* spp., *Proteus* spp., and *Escherichia* spp.) associated with OE. In addition, healthy dogs can also harbor the bacteria asymptomatically [6,10,11,12].

Microbiota refers to the living microorganisms present in a specific environment, such as the oral or gut microbiota, and in various parts of the body, including the oral cavity, intestines, skin, and ear canal [13]. On the other hand, the microbiome is the entirety of the genetic material of the microorganisms that form the microbiota, and also includes microbial structural elements, metabolites, and environmental conditions [14]. Previous research identifying the bacteria comprising the ear canal microbiota of dogs with healthy ears and OE has generally utilized culture-based methods or molecular analyses. Although cultural methods are considered the gold standard for isolating, identifying, and assessing the antimicrobial susceptibility of bacteria, they do not fully reveal microbial diversity, considering unculturable bacteria. In contrast, NGS-based microbiome analysis approaches, such as *16S rRNA* gene amplicon sequencing and shotgun metagenomic sequencing, provide more comprehensive and valuable data by detecting all bacteria in the microbiota [4,5,6,11,15,16].

We hypothesized that OE is associated with shifts in the ear microbial community, characterized by increased dominance of opportunistic pathogens and distinct antimicrobial resistance profiles compared to healthy ears. Combining bacterial culture-based methods and shotgun metagenomic profiling enables both species-level confirmation and comprehensive functional analysis. By integrating these approaches, this study provides complementary taxonomic and resistance-associated insights, thereby advancing current understanding of microbial dynamics in canine OE.

This study aimed to investigate ear swab samples from pet dogs in Kayseri, Türkiye, by characterizing the ear microbiota of healthy and OE-affected dogs using bacterial culture-based methods and shotgun metagenomic sequencing, together with read-based screening for antimicrobial resistance and virulence-associated genetic signatures.

## 2. Materials and Methods

### 2.1. Specimens

In this study, 200 ear swab specimens were collected from a single ear (either right or left) of 200 domestic dogs aged 1 to 10 years, brought to various pet clinics in Kayseri and the Department of Surgery, Faculty of Veterinary Medicine, Erciyes University, between 2023 and 2024. The dogs were classified as having OE (*n* = 100) or being healthy (*n* = 100). Diagnosis of OE was based on the presence of inflammation in the external ear canal, including erythema, oedema, pruritus, and otic discharge in clinical examination. Healthy dogs had no history or clinical signs of the disease. General clinical and demographic characteristics of the study population are presented in Table S1. The specimens were transported to the Microbiology Laboratory under cold chain conditions. All specimens were subjected to bacteriological analysis, and metagenomic analysis was performed on 10 randomly selected samples: five from dogs with OE and five from healthy dogs.

### 2.2. Bacterial Culture

Canine ear swab specimens were plated onto 7% sheep blood agar (Liofilchem 610188, Roseto degli Abruzzi, Italy) and MacConkey agar (Neogen NCM0017A, Lansing, MI, USA). The blood agar plates were incubated at 37 °C for 24–48 h under aerobic and microaerophilic (MGC AnaeroPack-MicroAero, GB-255AE, Tokyo, Japan) conditions, and for 24–96 h under anaerobic (MGC AnaeroPack-Anaero, GA-500ZE) conditions; MacConkey agar was incubated at 37 °C for 24–48 h under aerobic conditions. At the end of the incubation period, the colonies growing in Petri dishes were evaluated for macroscopic/microscopic features and biochemical tests including oxidase, catalase, coagulase, indole production, and hydrogen sulphide production. The pure cultures of the isolates were stored at −84 °C in Brucella Broth (Thermo Fisher Scientific, R452662, Waltham, MA, USA) containing 15% glycerol.

### 2.3. Microbiological Identification

#### 2.3.1. Matrix-Assisted Laser Desorption/Ionization Time-of-Flight Mass Spectrometry (MALDI-TOF MS)

A whole-cell sample was prepared from pure bacterial colonies as previously described [17]. Briefly, single colonies were applied as a thin film onto a MALDI target plate, overlaid with 1 µL of matrix solution, and allowed to air-dry at room temperature before insertion into the instrument. To determine all mass spectra, an Ultraflex II MALDI-TOF/TOF mass spectrometer (Bruker Daltonics, Billerica, MA, USA) equipped with a fully solid-state SmartBeam™ laser (Nd: YAG laser) operating at 100 Hz in positive linear mode (100 ns, 25 kV, and 2.2–20 kDa) under Flex Control software version 3.0 (Bruker Daltonics) control was used. According to the manufacturer’s specified criteria, score values ≥ 2.00, 1.70–1.99, and <1.70 were defined as correct species, low consistency, and inconsistency, respectively [18].

#### 2.3.2. 16S rRNA Gene Sequencing

The analysis was performed on isolates that could not be identified at the species level via MALDI-TOF MS. For this purpose, DNA was extracted from single bacterial colonies using the classical Single Cell Lysis Buffer (SCLB) protocol, which involves cell lysis, removal of cellular debris by centrifugation, and collection of the supernatant containing genomic DNA, as previously described [19]. PCR amplification of the *16S rRNA* gene was performed using universal 27F and 1492R primers, targeting a 1465 bp fragment as described by Lane et al. [20], followed by purification of the PCR products using a standard column-based cleanup kit to remove primers and nucleotides before sequencing. Chromatograms obtained from sequence analysis were evaluated and aligned using the Geneious (version 2025) program. The results were identified in the National Center for Biotechnology Information (NCBI), Basic Local Alignment Search Tool (BLAST) rRNA/ITS database, and, following the necessary procedures, were deposited in the National Institutes of Health GenBank [21].

### 2.4. Shotgun Metagenomic Sequencing

Shotgun metagenomic sequencing was performed to enable comprehensive and unbiased characterization of the microbial community, including bacterial, fungal, viral, and other microbial DNA present in the ear samples. The analyzed samples (*n* = 10) were categorized as follows: specimens from animals with OE were coded “O”, and “H” indicated samples from healthy animals. Genomic DNA from the samples was extracted using the commercial GeneJET Genomic DNA Purification Kit (Thermo Fisher Scientific, K0721, USA), following the manufacturer’s instructions. The concentrations of the obtained DNA samples were determined fluorometrically by the Qubit platform, and their purities were evaluated spectrophotometrically via a NanoDrop instrument (Thermo Fisher Scientific, Waltham, MA, USA). The purified DNA samples were then used as input for paired-end Illumina shotgun whole-genome sequencing (WGS), and raw sequencing reads were generated in FASTQ format.

### 2.5. Data Analysis

#### 2.5.1. Read Quality Control and Preprocessing

The raw sequencing reads were subjected to quality control and preprocessing before downstream analyses to minimise sequencing-related artefacts. Read quality was evaluated using FastQC [22], and sample-level quality metrics were summarised with MultiQC [23]. Adapter sequences and low-quality bases were removed using fastp [24] with standard trimming thresholds. Post-trimming read quality was re-evaluated using FastQC and MultiQC to verify data integrity and appropriateness in terms of subsequent analyses. After quality filtering, reads were aligned to the dog reference genome (*Canis lupus familiaris*; CanFam3.1/canFam3) and the human reference genome (*Homo sapiens*; GRCh38/hg38) by Bowtie2 [25]. Reads mapping to host reference genomes were removed, and only non-host reads were retained for downstream metagenomic analyses.

#### 2.5.2. Taxonomic Profiling and Abundance Estimation

Taxonomic classification of microbial reads was carried out using Kraken2 [26]. The PlusPF database (Version: 15 October 2025), comprising RefSeq bacterial, fungal, and protozoal genomes, was used as the reference. Classification was conducted in paired-end mode, and a minimum confidence threshold was applied to improve assignment reliability. Kraken2 classification outputs were subsequently processed with Bracken [27] to obtain species-level abundance estimates. Following taxonomic assignment, abundance tables were normalized on a per-sample basis to calculate relative abundances, ensuring that the totals summed to 100% for each sample.

#### 2.5.3. Alpha and Beta Diversity Analyses

Alpha diversity was evaluated at the species level by the Shannon and Simpson diversity indices, as performed in the vegan R package (v2.6-4) [28]. Due to the limited sample size and the absence of assumptions about data normality, differences between healthy and OE groups were investigated using the non-parametric Wilcoxon rank-sum test.

Beta diversity was quantified using Bray–Curtis dissimilarities calculated from species-level relative abundance tables. Ordination was done by principal coordinates analysis (PCoA). Group-level differences in microbial community composition were evaluated using permutational multivariate analysis of variance (PERMANOVA; adonis2) with 999 permutations [29], as performed in the vegan package, to evaluate the statistical significance of clustering patterns observed.

#### 2.5.4. Core Microbiome Definition

Core microbiome analysis was performed at the species level to define taxa consistently determined within each group analysed. Bracken-derived species abundance tables were utilized, and the prevalence of species was identified as the number of samples within a group in which a species was detected by at least one read [30]. A species was classified as part of the core microbiome if it was found in at least 60% of samples within the relevant group (i.e., ≥3 of 5 samples). This prevalence-based threshold was applied to reduce the influence of stochastic detection in small-sample datasets and to focus on stable community members. Core species were categorised as unique to the healthy group, unique to the OE group, or shared between the two groups. Group-level overlap patterns were summarised using a two-set Venn diagram, reporting the number of core species per category. For genus-level interpretation, the species-level core lists were additionally summarised at the genus level by aggregating core species under their respective genera, and the number of core species represented within each genus was calculated separately for the healthy core, otitis core, and shared core.

#### 2.5.5. Differential Abundance Screening

Species-level differential abundance was investigated with log2 fold changes in relative abundance between healthy and OE groups as an effect-size-based screening approach. Relative abundance tables were normalised to sum to 100% for each sample, and group-based mean relative abundances were calculated. To enable log-ratio computation in the presence of zero values, a small pseudo-count (1 × 10^−6^) was added before fold-change estimation. Species with an absolute log2 fold change ≥ 1 were considered to exhibit pronounced directional shifts and were visualised using directional bar plots. This analysis was interpreted descriptively, given the compositional nature of sequencing-derived relative abundance data [31].

#### 2.5.6. Hierarchical Clustering and Prevalence-Based Visualisation

Hierarchical clustering based on Bray–Curtis dissimilarities was done to examine further similarities among samples using species-level relative abundance profiles. An agglomerative clustering approach with average linkage (UPGMA) was applied to the Bray–Curtis distance matrix. The obtained distance structure was visualised as a clustered heatmap with dendrograms. For visualisation purposes, species abundances were log10-transformed to improve colour scaling, whereas Bray–Curtis dissimilarities were calculated using non-transformed relative abundance data [32]. To enhance interpretability, taxa were filtered for visualisation using a prevalence–abundance criterion: species were retained if they were detected in at least 30% of samples and had a relative abundance of 0.5% in at least one sample. Among retained taxa, the top 30 species according to total relative abundance were selected. This filtering was applied exclusively for visualisation and did not affect diversity or statistical analyses. Using the same filtered species set, prevalence was calculated as the percentage of samples in each group that contained a species [30]. Group-based prevalence and mean relative abundance were then jointly visualised using bubble plots.

#### 2.5.7. Antimicrobial Resistance Gene Profiling

Antimicrobial resistance (AMR) determinants were identified from paired-end metagenomic datasets (H1-H5 and O1-O5) using the Resistance Gene Identifier (RGI) read-mapping workflow (RGI-bwt; v6.0.2) against the Comprehensive Antibiotic Resistance Database (CARD) [33]. Host-depleted, quality-filtered read pairs were screened against CARD reference sequences with Bowtie2 in the RGI-bwt pipeline [25]. High-confidence detection criteria were used, including a minimum average coverage of 50%, at least 300 bp of aligned length, and a minimum of five mapped reads per AMR gene. AMR results were summarized using antibiotic resistance ontology (ARO) terms, including mapping support, coverage metrics, and CARD functional annotations such as resistance mechanisms, gene families, and related drug classes. AMR detection was considered evidence at the read level for resistance determinants rather than a direct indicator of phenotypic resistance.

#### 2.5.8. Virulence Factor Profiling

Virulence gene detection was carried out on host-depleted paired-end reads using a read-based protein alignment strategy against the Virulence Factor Database (VFDB) Set A [34]. For each sample, forward and reverse reads were aligned independently using DIAMOND blastx in sensitive mode, applying an E-value threshold of 1 × 10^−5^ and retaining the best hit per read [35]. Paired-end results were combined for each sample, and additional stringency was applied by retaining only matches with ≥90% amino acid identity and an alignment length of ≥25 amino acids. The identified virulence factors were considered genetic indicators of potential virulence, not proof of functional expression in vivo.

## 3. Results

### 3.1. Bacterial Culture

Ear swab specimens from dogs with OE and healthy dogs were positive in 91% and 80%, respectively. A total of 336 isolates were recovered, with 177 from dogs with OE and 159 from healthy dogs. A total of 263 isolates were identified at the species level via MALDI-TOF MS; however, the remaining 73 isolates (43 from dogs with OE and 30 from healthy dogs) had scores < 1.70 and could not be identified at the species level. The identification of these isolates was performed using *16S rRNA* gene sequencing; 60 isolates were defined at the species level and 13 at the genus level (eight *Bacillus* spp., two *Paenibacillus* spp., one *Corynebacterium* sp., one *Faecalicoccus* sp., and one *Streptomyces* sp.). The *16S rRNA* gene sequences of the isolates were deposited in GenBank under accession numbers PV688025-PV688027, PX069564, and PX760510-PX760578.

**Figure 1:**
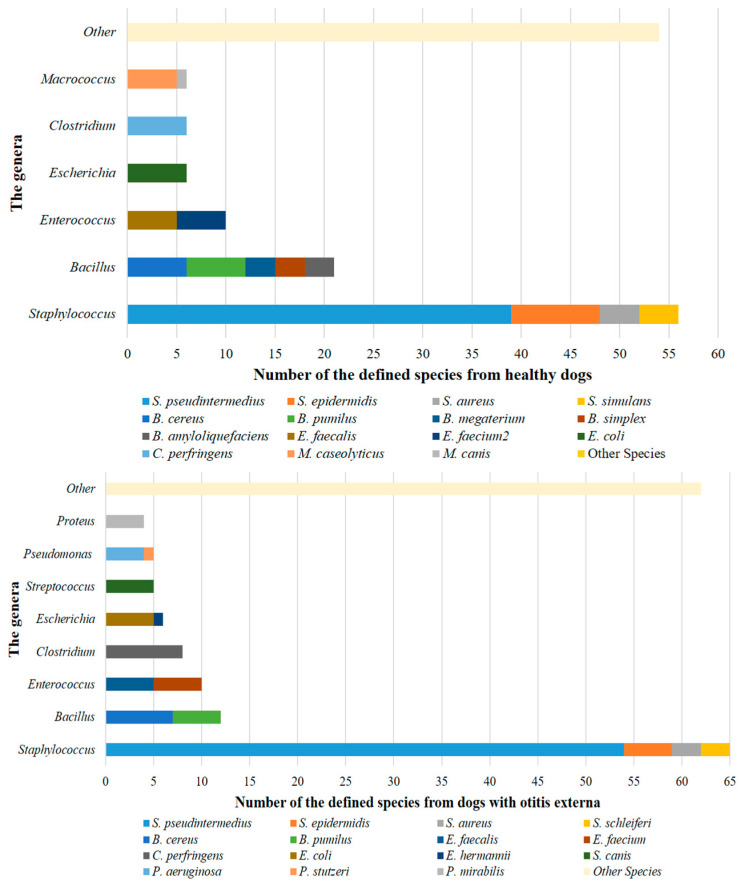
The most commonly identified bacteria in dogs with healthy and otitis externa.

In the present study, the isolates from both dogs with OE and healthy dogs were included in 26 different genera, yielding a total of 37 genera. Furthermore, the isolates from healthy dogs comprised 56 distinct species, while those from dogs with OE comprised 63 distinct species, resulting in a total of 97 species. Of the 336 isolates from dog ear specimens, 229 (68.2%) belonged to the genera *Staphylococcus* spp. (42.3%), *Bacillus* spp. (17.9%), and *Enterococcus* spp. (8%), respectively. The most frequently identified species were *Staphylococcus pseudintermedius* (27.7%), *Staphylococcus epidermidis* (4.2%), *Clostridium perfringens* (4.2%), *Bacillus cereus* (3.9%), *E. coli* (3.3%), and *Bacillus pumilus* (3.3%), respectively. In addition, the most commonly defined species in dogs with OE were *S. pseudintermedius* (30.5%), *C. perfringens* (4.5%), *B. cereus* (4%), *S. epidermidis* (2.8%), *E. coli* (2.8%), *B. pumilus* (2.8%), *Enterococcus faecalis* (2.8%), *Enterococcus faecium* (2.8%) and *Streptococcus canis* (2.8%), respectively; the most common species in healthy dogs were *S. pseudintermedius* (24.5%), *S. epidermidis* (5.7%), *E. coli* (3.8%), *C. perfringens* (3.8%), *B. cereus* (3.8%), and *B. pumilus* (3.8%), respectively (Figure 1 and Table S2).

### 3.2. Metagenomic Analysis

#### 3.2.1. Read Quality Control and Host Read Removal

Illumina shotgun sequencing generated 11.3–35.3 million paired-end reads for each sample in the dataset. Raw reads showed consistently high quality, with average read lengths of 146–151 bp and stable GC content (41–45%) across samples. Adapter and quality trimming with fastp retained more than 97% of reads per sample, with minimal changes in read length distribution and GC content. No systematic differences in sequencing depth or read quality were observed between healthy and OE groups, supporting the technical comparability of samples for downstream analyses. Following quality filtering, paired-end reads were mapped to the combined dog (*Canis lupus familiaris*, CanFam3.1) and human (*Homo sapiens*, GRCh38) reference genomes using Bowtie2 to remove host-associated sequences. Overall host alignment rates ranged from 42.8% to 99.69% across samples, with healthy samples showing alignment rates between 42.8% (H4) and 99.6% (H3), and OE samples ranging from 97.98% (O4) to 99.69% (O2). Reads not aligning to host reference genomes were retained as non-host sequences for downstream analyses. The proportion of non-host read pairs varied from approximately 5% to 57% across samples. In absolute terms, the number of retained non-host read pairs ranged from 0.50 million to 23.01 million per sample. Among healthy samples, non-host read counts varied between 1.93 million (H5) and 23.01 million (H4), whereas OE samples retained between 0.50 million (O2) and 2.52 million (O1) non-host read pairs. All retained non-host reads were subsequently used for downstream taxonomic, diversity, and functional analyses.

#### 3.2.2. Taxonomic Profiling and Abundance Estimation

Species-level taxonomic profiling defined a total of 5579 microbial taxa across all samples, based on relative abundance estimates (Table S3). The composition of the microbial community was uneven across samples, with a limited number of taxa accounting for an important proportion of the total relative abundance.

In the healthy samples, microbial communities were characterized by higher relative abundances of *Bacteroides fragilis* (15.8%), *Ezakiella coagulans* (8.2%), *Proteus mirabilis* (7.4%), *Escherichia coli* (4.9%), and *Acinetobacter lwoffii* (4.2%). In contrast, *Staphylococcus pseudintermedius* was the dominant species in the OE samples (38.7%), with additional contributions from *Pasteurella canis* (9.3%), *Malassezia restricta* (8.2%), and *Fusarium oxysporum* (6.8%). *Cutibacterium acnes* was detected in both clinical groups at lower relative abundances (healthy: 2.7%; OE: 1.4%).

**Figure 2:**
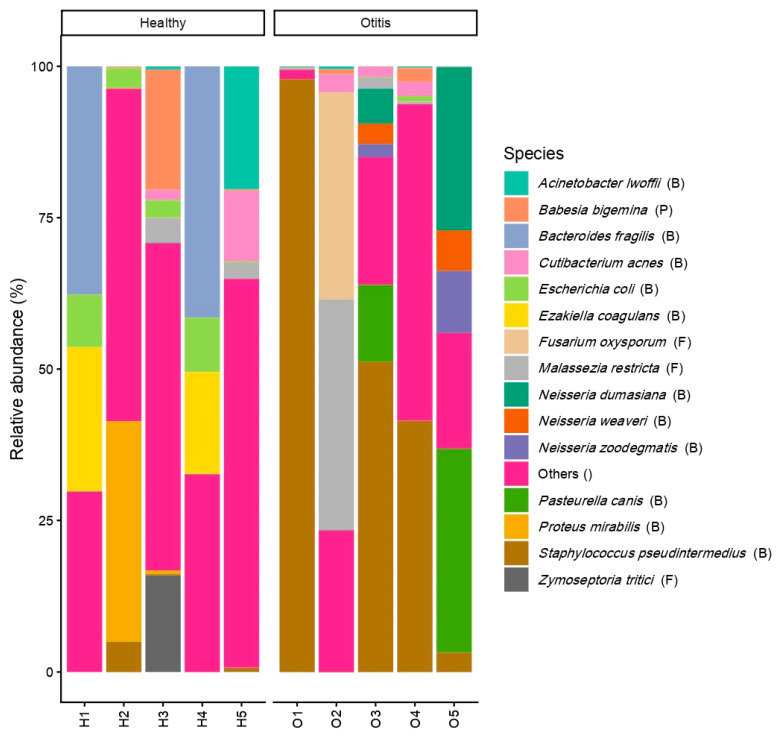
Relative abundance of the top 15 microbial species across healthy and otitis externa canine ear specimens. Letters in parentheses indicate organismal groups: B, bacteria; F, fungi; P, parasites.

A stacked bar plot showing the relative abundances of the top 15 species across all samples illustrated pronounced inter-individual variability within both healthy and OE groups, while lower-abundance taxa collectively accounted for a substantial fraction of the microbial signal (Figure 2). Detailed species-level relative abundance values for all detected taxa are provided in Table S3.

#### 3.2.3. Alpha and Beta Diversity Analyses

**Figure 3:**
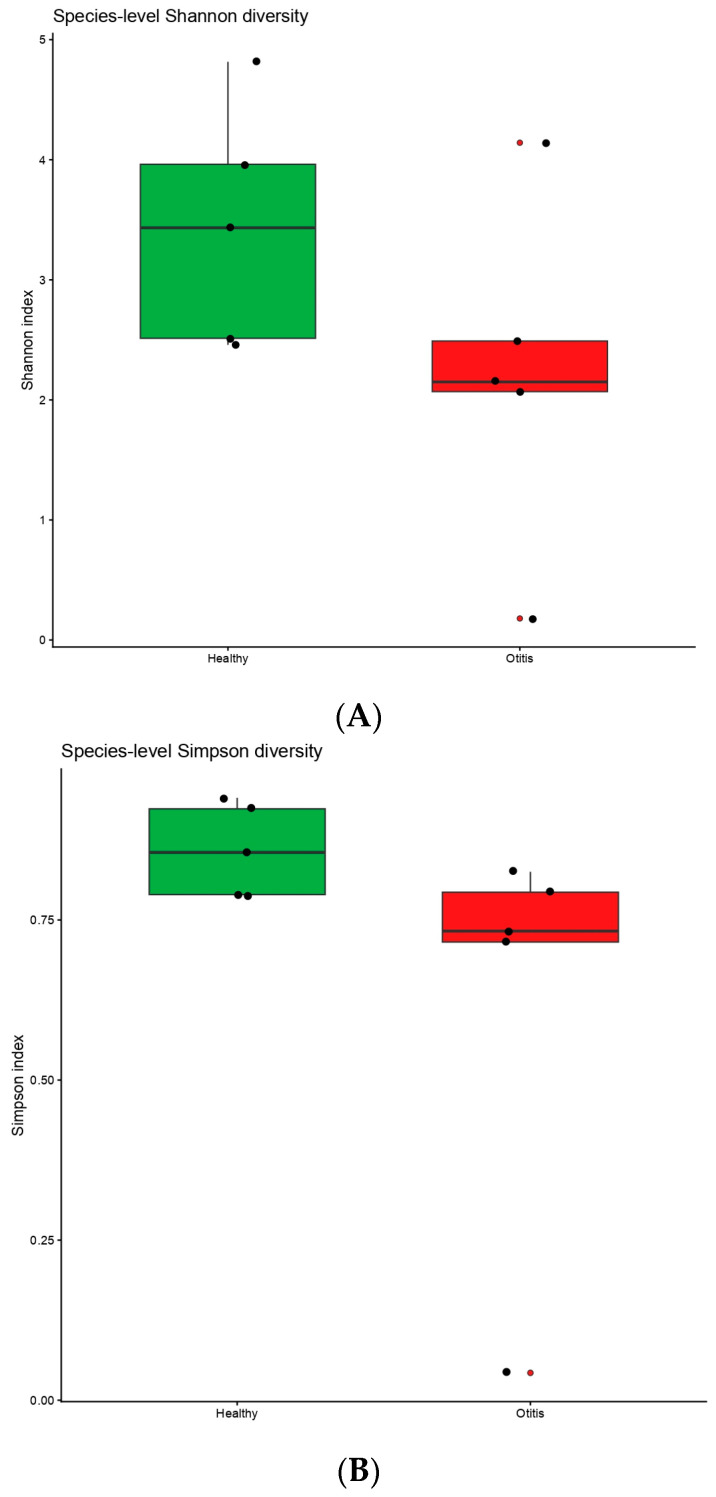

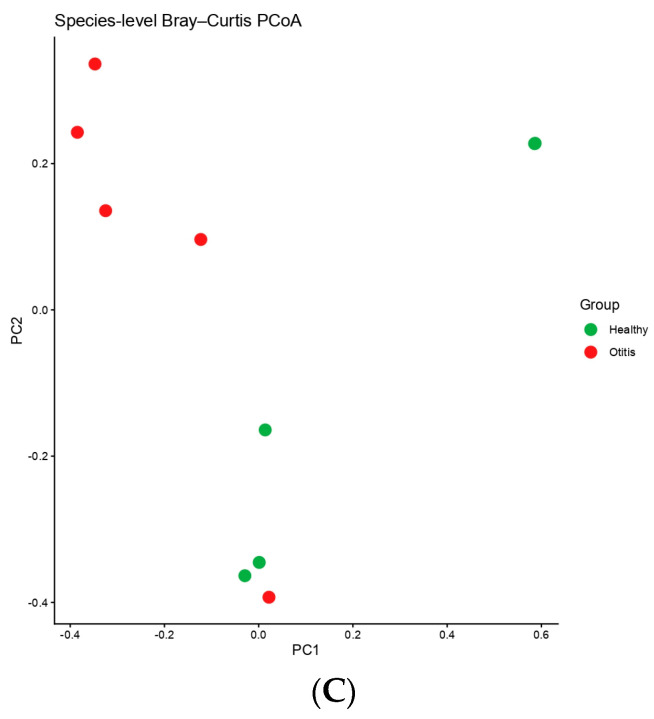
(**A-C**). Alpha diversity (Shannon, (**A**); Simpson, (**B**)) and beta diversity (Bray-Curtis PCoA, (**C**)) comparisons between healthy and OE samples. Group differences were assessed using the Wilcoxon rank-sum test (Shannon: W = 20, *p* = 0.151; Simpson: W = 21, *p* = 0.095) and PERMANOVA (R^2^ = 0.21, F = 2.13, *p* = 0.019).

Species-level alpha and beta diversity analyses were performed to compare microbial community structure between healthy and OE samples (Figure 3A–C). Shannon diversity values in healthy samples ranged from 2.46 to 4.82, whereas OE samples showed a broader range of 0.18 to 4.14; similarly, Simpson diversity values ranged from 0.79 to 0.94 in healthy samples and from 0.04 to 0.83 in OE samples (Table S4). Group-level differences in alpha diversity were evaluated using the Wilcoxon rank-sum test, which did not reveal a statistically significant difference between healthy and OE groups for either the Shannon index (W = 20, *p* = 0.151) or the Simpson index (W = 21, *p* = 0.095). Species-level beta diversity was assessed using Bray–Curtis dissimilarities and visualised by principal coordinates analysis (PCoA) (Figure 3C), with healthy samples positioned on the positive side of the first principal coordinate (PC1) and all OE samples located on the negative side. The significance of group-level differences in community composition was further evaluated by PERMANOVA, which indicated a significant effect of clinical status on species-level microbial composition (R^2^ = 0.21, F = 2.13, *p* = 0.019).

#### 3.2.4. Core Microbiome

**Figure 4:**
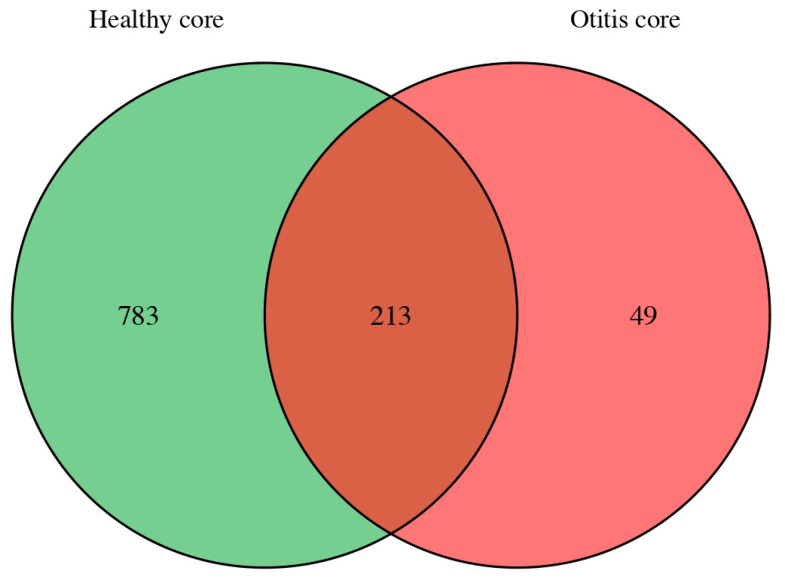
Core microbiome composition at the species level in healthy and otitis externa canine ear specimens.

Core microbiome analysis was conducted at the species level using a prevalence threshold of ≥60% to identify taxa consistently detected within each clinical group. Based on the Venn-based classification of species meeting this criterion, 783 species were identified as healthy-specific core members (core in healthy but not in otitis), 49 species were classified as otitis-specific core members (core in otitis but not in healthy), and 213 species met the core criterion in both groups and were therefore classified as shared core taxa (Figure 4). Accordingly, the total number of core species meeting the ≥60% prevalence threshold was 996 in the healthy group (783 healthy-specific + 213 shared) and 262 in the OE group (49 otitis-specific + 213 shared), indicating that stable community membership was largely group-dependent and that the healthy group exhibited a substantially broader core community structure than the OE group. For genus-level interpretation, the species-level core lists were additionally summarised at the genus level. The healthy-specific core (783 species) encompassed 311 genera and was most prominently represented by *Corynebacterium* spp. (40 species), *Psychrobacter* spp. (27), *Streptococcus* spp. (24), and *Acinetobacter* spp. (21), followed by *Pseudomonas* spp. and *Stenotrophomonas* spp. (16 each). In contrast, the otitis-specific core (49 species) comprised 41 genera and was comparatively restricted, with *Staphylococcus* spp. (5 species) and *Neisseria* spp. (3) representing the most recurrent genera. The shared core (213 species) spanned 111 genera and was most frequently represented by *Streptococcus* spp. (11 species), followed by *Paracoccus* spp., *Sphingomonas* spp., and *Neisseria* spp. (8 each), and *Corynebacterium* spp. (7). A detailed species-level list with prevalence values and group classification is provided in Table S5, and the genus-level summary is provided in Table S6.

#### 3.2.5. Differential Abundance

**Figure 5:**
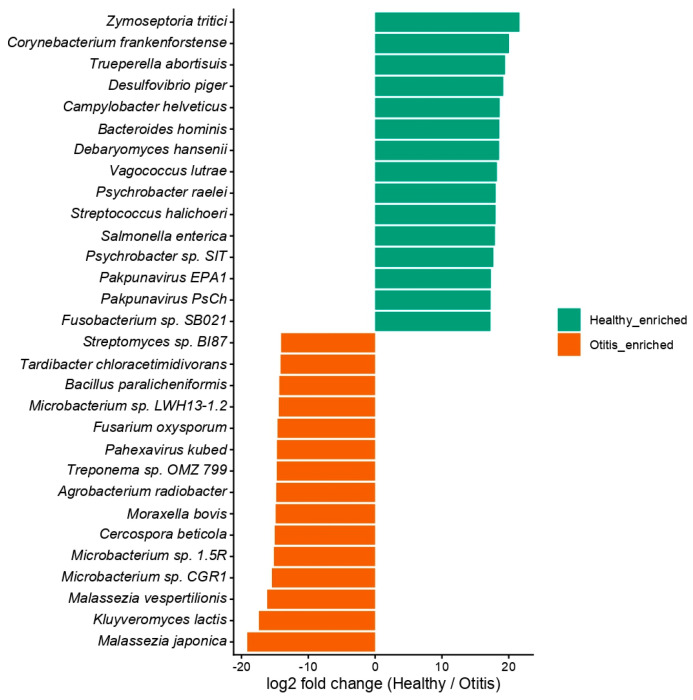
Differentially abundant species between healthy and OE groups (differential abundance output; effect-size ranking).

Species-level differential abundance screening based on log2 fold change identified species with the largest directional shifts between healthy and OE groups. For visualisation, only the species with the highest absolute effect sizes were retained, specifically the top 20 healthy-enriched and top 20 Otitis-enriched taxa ranked by log2 fold change (Figure 5). The resulting profile comprised a mixture of bacterial, fungal, and viral taxa, including healthy-enriched species such as *Zymoseptoria tritici*, *Corynebacterium frankenforstense*, *Trueperella abortisuis*, *Desulfovibrio piger*, and *Campylobacter helveticus*, and Otitis-enriched species such as *Malassezia japonica*, *Kluyveromyces lactis*, *Malassezia vespertilionis*, *Microbacterium* sp., *Cercospora beticola*, and *Moraxella bovis* (Figure 5).

#### 3.2.6. Hierarchical Clustering and Prevalence-Based Visualisation

**Figure 6:**
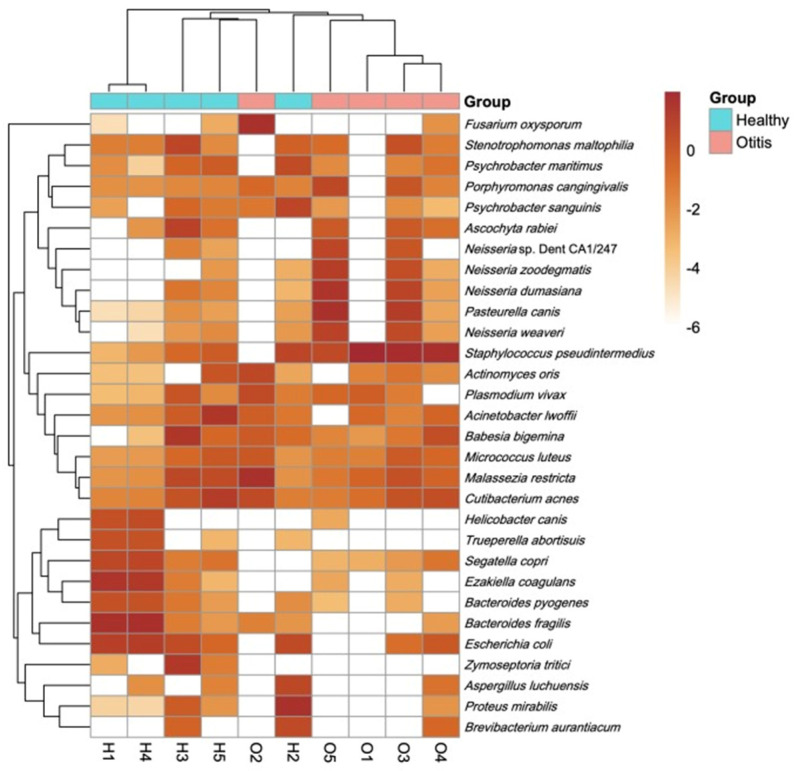
Unsupervised hierarchical clustering heatmap of species-level relative abundances in healthy and otitis externa canine ear specimens.

Species-level relative abundance profiles were filtered for visualisation and used to compute Bray–Curtis dissimilarities, followed by agglomerative hierarchical clustering with average linkage. The resulting clustered heatmap resolved two primary sample groupings: healthy samples H1 and H4 formed a close pair, clustering adjacent to H3 and H5, whereas the remaining samples (including O2, H2, and O1-O5) grouped on a separate branch (Figure 6). Within the filtered species set displayed in the heatmap, *S. pseudintermedius*, *P. canis*, multiple *Neisseria* taxa (including *N. dumasiana*, *N. zoodegmatis*, and *N. weaveri*), *M. restricta*, *P. mirabilis*, and *Fusarium oxysporum* were among the taxa contributing to the observed between-sample structure (Figure 6).

#### 3.2.7. AMR and Virulence Genes

Antimicrobial resistance gene profiling (CARD/RGI-bwt) yielded high-confidence AMR gene matches across the dataset, with marked variation in mapped-read support among individual samples (Table S7). In the healthy group, the highest mapped-read burdens were recorded in H1 and H4, driven primarily by multidrug efflux-associated gene families and tetracycline target-protection determinants. In H1, the most significant contributors among the high-confidence gene-family summaries were the resistance-nodulation-cell division (RND) antibiotic efflux pump (All Mapped Reads = 5552) and major facilitator superfamily (MFS) antibiotic efflux pump (All Mapped Reads = 5105), followed by tetracycline-resistant ribosomal protection proteins (All Mapped Reads = 2768) and *Erm 23S rRNA* methyltransferases (All Mapped Reads = 1667); additional high-confidence signals included pmr phosphoethanolamine transferase (All Mapped Reads = 920) and *CepA* beta-lactamase (All Mapped Reads = 846). In H4, tetracycline-resistant ribosomal protection proteins (All Mapped Reads = 3111), *Erm 23S rRNA* methyltransferases (All Mapped Reads = 1244), and CepA beta-lactamase (All Mapped Reads = 952) were prominent, together with pmr phosphoethanolamine transferase (All Mapped Reads = 822) and additional beta-lactamase signals including *CfxA* beta-lactamase (All Mapped Reads = 326). Within the OE group, high-confidence hits were sample-dependent, with O1 showing the most pronounced mapped-read support among OE samples. In O1, methicillin-resistant PBP2-associated determinants were detected with high mapped-read counts (e.g., *mecR1* with All Mapped Reads = 381 and methicillin-resistant PBP2 with All Mapped Reads = 956), alongside tetracycline-resistant ribosomal protection protein (All Mapped Reads = 434) and an MFS antibiotic efflux pump signal represented by mef(E) (All Mapped Reads = 76). In contrast, O2 had no high-confidence hits reported in the “High-confidence hits” section. Other OE samples carried lower but detectable high-confidence signals, including *mef*(E) in O3 (All Mapped Reads = 12) and in O4 (All Mapped Reads = 26), as well as *sul2* in O4 (All Mapped Reads = 8). At the drug-class and mechanism levels, the dataset contained high-confidence determinants associated with beta-lactams, tetracyclines, aminoglycosides, macrolides, and sulfonamides, with gene-family patterns summarised in Table 1, including beta-lactamases (e.g., *CepA*, *CfxA*, *CTX-M*), tetracycline target protection (*tet* family), aminoglycoside-modifying enzymes (AAC/APH/ANT), Erm family methyltransferases, RND/MFS efflux pumps, and sul genes (Table 1; Figures S1 and S2; Table S7).

Virulence factor profiling (VFDB Set A/DIAMOND blastx) identified virulence-associated genes across all samples, and group-level category summaries are provided in Table 2. Categories classified as adhesion/surface-associated factors included detections mapped to representative genes such as *spa*, *clfA*, *clfB*, and *fnbA*. Immune evasion-related factors comprised capsule-associated genes and surface antigens. Tissue damage/invasion included extracellular proteases and invasion-associated enzymes. Secretion-associated factors included secretion system-associated proteins. A fifth category comprised conserved virulence homologs, which were reported to show similar patterns between groups in the category-level summary (Table 2; Figures S3 and S4; Table S8).

Enrichment was determined by comparing group-level mean relative abundances of virulence-associated reads, calculated as the proportion of reads mapping to each virulence category relative to total VFDB-mapped reads per sample, followed by averaging within healthy and OE groups.

## 4. Discussion

Otitis externa is among the major diseases affecting pet dogs worldwide. Many zoonotic and antimicrobial-resistant pathogenic bacteria contribute to disease pathogenesis, and determining their distribution is critical for effective disease management. In addition, healthy pet dogs may harbour these bacteria in their ear canal microbiota; therefore, identifying the bacteria that comprise the ear microbiota in both dogs with OE and healthy dogs is important [12,36].

The bacterial culture-based studies investigating the ear canal microbiota of dogs with OE and healthy dogs have shown differences in the bacteria identified. Indeed, according to the literature review, *S. pseudintermedius*, a pathogenic microorganism responsible for many infections, including OE in veterinary medicine, has been the most frequently identified species (3.6–54.7%) in recent years among dogs with OE cases [11,12,36,37,38,39,40,41]. Furthermore, other bacteria most commonly defined in dogs with OE are: other coagulase-positive and negative staphylococci (*S. intermedius* 18.4–73.9%, *S. aureus* 3.9–44.1%, *S. epidermidis* 5.7–22.1%, *S. schleiferi* 2.9–9.2%, and *Staphylococcus* spp. 26.7–59%), *Pseudomonas* (*P. aeruginosa* 2.9–60%, and *Pseudomonas* spp. 15–19.9%), *Proteus* (*P. mirabilis* 3.3–25%, and *Proteus* spp. 4.3–14.4%), *E. coli* (1.1–14.1%), streptococci (*Str. canis* 2.5–29.9%, and *Streptococcus* spp. 5–18%), *Enterococcus* spp. (1.86–14%), and *Bacillus* spp. (18.6%) [10,11,12,36,37,38,39,40,41,42,43,44,45,46,47,48,49,50]. Compared with OE, few studies have characterized the ear canal microbiota of healthy dogs using bacterial culture-based methods, and the available data are limited. In these studies, the primary bacterial agents identified were staphylococci (*S. aureus* 5–31.5%, *S. intermedius* 9.5–24.2%, *Staphylococcus* spp. 20.8–46.7%), *Bacillus* spp. (12.3–23%), *Proteus* spp. (3.9–8.2%), *Streptococcus* spp. (16.4%), *P. aeruginosa* (8.2%), *E. coli* (6.8%), and *Micrococcus* spp. (4–6.2%) [8,43,51,52].

In our study, the most frequently defined species in the ears of dogs with OE was *S. pseudintermedius* at 30.5%, followed by *C. perfringens* at 4.5%, *B. cereus* at 4%, and *S. epidermidis*, *E. coli*, *B. pumilus*, *E. faecalis*, *E. faecium*, and *Str. canis* each at 2.8%. *Staphylococcus* spp. was found in 43.5%, with *S. aureus*/*S. schleiferi* each at 1.7%, *Bacillus* spp. at 16.9%, and *P. aeruginosa*/*P. mirabilis* each at 2.3%. In addition, the most commonly identified species in healthy dogs were *S. pseudintermedius* (24.5%), *S. epidermidis* (5.7%), and *E. coli*, *C. perfringens*, *B. cereus*, and *B. pumilus* (3.8% each). *Staphylococcus* spp., *Bacillus* spp., *Streptococcus* spp., *S. aureus*, *Micrococcus luteus*, and *P. mirabilis* were detected at rates of 40.9%, 18.9%, 3.1%, 2.5%, 1.9%, and 1.3%, respectively (Figure 1, Table S2). Compared with previous studies, our results are generally consistent with the literature. The identification of *S. pseudintermedius* at a rate of 30.5% in cases of OE supports a strong association between this microorganism and the disease. Likewise, the determination of *Staphylococcus* spp. at a rate of 43.5% is consistent with studies reporting that staphylococci are among the predominant bacterial agents in canine OE. However, there are some minor differences from the literature. Indeed, the relatively lower identification rates of *P. aeruginosa* and *P. mirabilis* (2.3%) suggest that these microorganisms play a more significant role in chronic disease. Furthermore, the presence of *Bacillus* spp. at a rate of 16.9% shows that environmental agents may form the canine ear canal microbiota as opportunistic microorganisms. Among ear specimens from healthy dogs, the detection of *S. pseudintermedius*, *Staphylococcus* spp., and *Bacillus* spp. at rates of 24.5%, 40.9%, and 18.9%, respectively, supports the idea that these microorganisms are part of the normal ear microbiota, may increase proportionally in cases of OE (except for *Bacillus*), and may exacerbate or prolong inflammatory reactions in the ear [53].

The ear canal microbiome is responsible for many important functions, including maintaining local immune balance, and disruptions or changes in this microbial community are associated with the development of canine OE. Shotgun metagenomic sequencing enables comprehensive, culture-independent characterization of microbial communities at high taxonomic resolution while also providing a range of information, including antimicrobial resistance profiles and the presence of virulence genes. The approach has recently been applied in human and veterinary microbiome studies to reveal dysbiosis-related diseases and host–microorganism interactions [4,15,54].

The current study used shotgun metagenomic sequencing to characterise microbial communities in ear swab specimens from dogs with healthy ears and OE, with additional read-based screening of AMR and virulence-associated genetic signatures. The sequencing output was uniformly high across the dataset (11.3–35.3 million paired-end reads per sample with >97% read retention after trimming), indicating that downstream inference was not limited by raw read quality. A key feature of the dataset was the large and variable host background after host read removal, compared with the combined canine and human reference genomes. In the OE group, host alignment was consistently high (97.98–99.69%) and microbial yield was accordingly low (0.50–2.52 million non-host read pairs). This technical constraint is important because reduced microbial depth can increase stochastic non-detection of low-abundance taxa and can attenuate the sensitivity of read-based functional mapping. In addition, low-biomass microbiome datasets are particularly vulnerable to reagent and laboratory contamination, which can disproportionately affect samples with low endogenous microbial DNA. Therefore, conservative interpretation and the inclusion of negative controls are widely recommended when applying sequencing-based approaches to low-biomass settings [55]. Within these constraints, species-level profiling detected 5579 microbial taxa across all samples. It revealed marked inter-individual heterogeneity, with a small number of taxa accounting for a substantial fraction of within-sample relative abundance. Several OE samples were dominated by *S. pseudintermedius*, with additional contributions from microorganisms including *P. canis* and *Malassezia* spp. This pattern is compatible with sequencing-based studies that describe altered otic microbiota in canine OE, highlighting *Staphylococcus* spp. and *Malassezia* spp. as commonly detected and clinically relevant taxa in affected ears. In particular, Korbelik et al. [56,57] reported distinct bacterial community profiles in OE compared with clinically healthy dogs, and similarly described differences in otic mycobiota in OE, with *Malassezia* spp. representing a prominent component of the canine ear mycobiome. Tang et al. [4] noted that the most abundant microorganisms in the infected ears were *Malassezia pachydermatis* (48.5%) and *S. pseudintermedius* (45.6%). Bradley et al. [15] found that similar taxa were present between otic and control dogs, but the relative abundance varied in each group, and *Staphylococcus* spp. was the most abundant taxon across all groups. Borriello et al. [53] studied the cerumen microbial community between healthy and otitis-affected dogs, and *Staphylococcus* spp. and *Pseudomonas* spp. were detected to be the bacterial genera responsible for most distances between the groups. Kasai et al. [5] determined that the otic community composition differed compared to the healthy control group, with significantly higher relative abundance of the phylum *Firmicutes* and the genus *Staphylococcus*. Saengchoowong et al. [6] also reported that the external ear canal harbours a diverse bacterial community and that *Staphylococcus* spp. are frequently detected in both healthy and otitis-associated contexts, supporting the interpretation that disease-relevant taxa may include organisms that are also part of baseline ear canal ecology. In the present dataset, *C. acnes* was detected at lower abundance across both clinical categories, consistent with the notion that some skin-associated organisms may be repeatedly detectable in ear swab metagenomes without implying disease specificity. On the other hand, in our study, the predominant microorganisms in healthy samples were *B. fragilis*, *E. coagulans*, and *P. mirabilis*. Compared to other studies, some differences are observed in the predominant microbial communities that form healthy canine ear microbiomes [4,5,6,15,53]. This is thought to be due to factors such as dog breed, age, sex, sample size, methodology, etc., as these variables can affect ear anatomy, immune function, cerumen production, and consequently the composition and abundance of microbial communities. Additionally, differences in study design and analytical methods may also contribute to variations in microbial detection between studies.

Alpha diversity metrics varied widely across samples, with some OE samples exhibiting extremely low-diversity profiles consistent with strong dominance by one or a few taxa. Bray–Curtis ordination suggested a directional separation pattern between OE and healthy samples along the first principal coordinate, and PERMANOVA indicated that clinical status explained a measurable fraction of compositional variance in this cohort (R^2^ = 0.21; *p* = 0.019). However, given the small cohort size and the substantial heterogeneity in effective microbial read depth driven by host background, these ordination and distance-based results should be interpreted as descriptive of the present dataset structure rather than as definitive evidence of population-level separation. In addition, our findings are parallel with previous studies reporting reduced alpha diversity, increased dominance by specific taxa, and partial, non-discrete beta-diversity separation between healthy and otic ear microbiota [5,53].

Core microbiome inference provided a complementary perspective on taxa that were repeatedly detectable across individuals. Using a ≥60% prevalence threshold, we identified 783 healthy-specific core species, 49 otitis-specific core species, and 213 shared core species (core in both groups). At the genus level, the healthy-specific core was taxonomically broad and enriched for commensal-associated genera such as *Corynebacterium* spp., *Psychrobacter* spp., and *Streptococcus* spp., whereas the otitis-specific core was comparatively restricted and most consistently represented by *Staphylococcus* spp. and *Neisseria* spp. Ecologically, an apparent reduction in core membership and contraction of genus-level breadth may be consistent with community destabilisation and restructuring associated with disease. However, differences in sequencing yield and host DNA background can also influence detectability, particularly for low-abundance taxa. This consideration is especially relevant for the otitis samples in the present dataset, which showed uniformly high host alignment and lower non-host read yields, conditions that can preferentially obscure rare community members. Accordingly, differences in core size and composition observed in this pilot dataset are most appropriately interpreted as a composite biological and technical signal, and should be validated in larger cohorts with more uniform microbial sequencing depth and the inclusion of dedicated low-biomass and negative controls.

The fold-change-based differential abundance screening identified taxa with the largest directional shifts in mean relative abundance, including multiple *Malassezia* taxa, which were enriched in otitis samples. The prominence of *Malassezia* spp. is consistent with the recognised role of yeasts in canine OE and with sequencing-based descriptions of otic mycobiota in affected dogs [57]. At the same time, effect-size screening of relative abundance profiles should be interpreted cautiously because microbiome readouts are compositional and can yield unstable or method-dependent differential signals, particularly in cases of sparsity and low counts. Gloor et al. [31] emphasised that sequencing-derived microbiome datasets are inherently compositional, motivating analytical approaches that explicitly account for this property. Methods such as Analysis of Compositions of Microbiomes (ANCOM) [58] and ANCOM with Bias Correction (ANCOM-BC) [59] were developed to improve differential abundance inference under compositional constraints. Extensive comparative evaluations have shown that different differential abundance tools can produce materially different results across datasets, reinforcing the need for method-aware interpretation and validation [60]. In this context, the fold-change signals in the present study are most appropriately framed as hypothesis-generating candidates for targeted confirmation.

Read-based functional screening provided additional clinically oriented context by surveying AMR and virulence-associated genetic signatures directly from host-depleted reads. CARD/RGI-bwt mapping yielded high-confidence AMR hits with strong sample-to-sample variability; the highest mapped-read burdens were observed in healthy-coded samples H1 and H4, mainly driven by efflux-associated families and tetracycline target-protection determinants, while one OE sample (O1) showed mec-associated determinants (*mecR1* and methicillin-resistant PBP2) alongside tetracycline ribosomal protection and an MFS efflux signal represented by *mef(E)*. The detection of mec-associated signatures in an OE sample is biologically plausible, given that methicillin-resistant *S. pseudintermedius* has been reported from canine OE, including studies that document *mecA*-positive isolates and multidrug resistance patterns in clinical contexts [61,62]. Nonetheless, read-level detection represents genetic potential and does not establish phenotypic resistance, genomic context, or expression, particularly in mixed communities; therefore, these results should be interpreted as supporting evidence to prioritise confirmatory culture-based susceptibility testing and/or assembly informed resistome reconstruction rather than as direct proxies for clinical resistance.

Virulence factor screening against VFDB Set A suggested category-level representation of adhesion/surface-associated factors and immune interaction signatures in OE samples, including loci such as *spa*, *clfA*, *clfB*, and *fnbA*. Mechanistically, enrichment of adhesion- and immune-evasion-associated signatures is coherent with pathogen-dominated states in inflamed tissue, where persistence and host interaction traits may be selectively advantageous. However, as with AMR profiling, read-based virulence screening reflects genomic potential and homology to reference sequences rather than demonstrated in vivo expression or causal contribution to disease. Consequently, future work would benefit from integrating targeted validation (e.g., qPCR panels for selected loci), isolate genomics, and clinical metadata to bridge the gap between metagenomic potential and functional or clinical relevance. In addition, to our knowledge, no comprehensive shotgun metagenomic studies have yet explored antimicrobial resistance (AMR) and virulence gene profiles in ear specimens from both healthy dogs and those with OE. Therefore, this study addresses that knowledge gap.

In the present study, culture-based methods allowed species-level identification of cultivable bacteria in all 200 samples, providing a foundation for understanding the dominant bacterial taxa. Shotgun metagenomic sequencing, performed on a subset of representative samples, complemented these findings by revealing additional uncultivable taxa and functional genetic potential, including antimicrobial resistance and virulence-associated genes. Together, these approaches provide a more comprehensive view of the canine ear microbiota than either method alone.

Several limitations frame the interpretation of this study. The cohort size is small, which restricts generalizability, while the consistently high host background in OE samples reduces effective microbial depth and increases the likelihood of stochastic non-detection and compositional instability for low-count taxa.

In low-biomass contexts, contamination control is also a central methodological concern, and future studies should incorporate and report extraction blanks and library negatives and implement contamination assessment strategies, as recommended in the low-biomass microbiome literature [55]. These constraints notwithstanding, the present dataset demonstrates that microbial signal can be recovered from host-dominated ear swab metagenomes and that species-level profiling can reveal inter-individual heterogeneity and pathogen-dominated configurations consistent with prior sequencing-based work in canine OE [6,56].

## 5. Conclusions

The current study’s cultural analysis of ear samples from healthy and OE-affected dogs showed that *S. pseudintermedius* was the most commonly identified species in both groups. Its presence in both healthy and infected ears indicates it is a typical part of the normal dog ear microbiota. However, its higher prevalence in OE cases suggests that changes in the microbial community could play a role in the disease process. Bacterial culture-based results highlight the polymicrobial nature of the dog’s ear canal and provide a foundational understanding of bacterial communities in health and disease. Complementing these results, species-level profiling of canine ear swab metagenomes revealed heterogeneous, individual-specific community structures and pathogen-dominated profiles in multiple OE samples, most prominently driven by *S. pseudintermedius*, together with additional bacterial and fungal signatures. Read-based functional screening further indicated sample-dependent AMR and virulence-associated genetic signals, supporting shotgun metagenomics as a hypothesis-generating framework for ear-canal microbiome research in low-biomass settings. Collectively, these findings justify larger, clinically annotated studies incorporating rigorous low-biomass controls and confirmatory culture or targeted molecular validation to strengthen organism-level attribution and clarify the clinical relevance of resistance and virulence determinants.

## Figures and Tables

**Table 1:** Major AMR gene families and associated antibiotic classes detected by RGI-bwt.

AMR Gene Family/Mechanism	Associated Antibiotic Class	Pattern in Healthy Samples	Pattern in Otitis Samples
Beta-lactamases (e.g., *CepA*, *CfxA*, *CTX-M*)	Beta-lactams	Detected in multiple samples with high read counts (notably H1, H4)	Strong signal in O1; limited detection in other samples
Tetracycline target protection (tet family)	Tetracyclines	High read counts in selected healthy samples	Present in O1 and O5
Aminoglycoside-modifying enzymes (AAC/APH/ANT)	Aminoglycosides	Low to moderate detection	Detected in a limited number of samples
Erm family (*23S rRNA* methyltransferases)	Macrolide–Lincosamide–Streptogramin	Prominent in H1 and H4	Low-level detection
RND/MFS efflux pumps	Multidrug resistance	Widespread with high mapped read counts	Present, generally with lower read counts
Sulfonamide resistance genes (*sul*)	Sulfonamides	Detected in some healthy samples	Low-level detection (e.g., O4)

**Note:** Terms such as “high” or “low” refer to **comparative read mapping patterns observed in the RGI-bwt report** and do not represent normalized or quantitative abundance estimates.

**Table 2:** Enrichment of virulence-associated genes detected by VFDB.

Virulence Category	Representative Virulence Genes (VFDB Set A)	Enrichment Pattern
Adhesion/surface-associated factors	*spa*, *clfA*, *clfB*, *fnbA*	Enriched in Otitis
Immune evasion-related factors	capsule-associated genes, surface antigens	Enriched in Otitis
Tissue damage/invasion	extracellular proteases, invasion-associated enzymes	Enriched in Otitis
Secretion-associated factors	secretion system-related proteins	Enriched in Otitis
Conserved virulence homologs	housekeeping-associated virulence homologs	Similar between groups

## Data Availability

The original contributions presented in this study are included in the article. Further inquiries can be directed to the corresponding author.

## Supplementary Materials

The following supporting information can be downloaded at https://www.mdpi.com/article/10.3390/vetsci13030250/s1: Table S1. General clinical and demographic characteristics of the study population; Table S2. Frequencies of the bacteria defined from ear swab specimens; Table S3. Species-level relative abundance matrix derived from shotgun metagenomic sequencing; Table S4. Species-level Alpha and Beta diversity metrics for individual samples; Table S5. Species-level core microbiome composition of healthy and otitis samples; Table S6. Genus-level summary of species-level core microbiome lists; Table S7. High-confidence AMR gene mapping results generated by the RGI-bwt read-mapping workflow against the Comprehensive Antibiotic Resistance Database (CARD) for each metagenomic sample (H1–H5 and O1–O5); Table S8. VFDB Set A virulence factor screening summary for each metagenomic sample based on DIAMOND blastx alignments of host-depleted paired-end reads.
