# Supplementary material for: Culture and Metagenomic Insights into the Ear Microbiota in Dogs with Healthy Ears and Otitis Externa
Source: Vet Sci. 2026 Mar 6;13(3):250. doi: 10.3390/vetsci13030250 (PMC13030325; doi:10.3390/vetsci13030250)
Supplement: Supplementary file 1 [file vetsci-13-00250-s001.zip › Table S1.pdf]

Table S1. General clinical and demographic characteristics of the study population

Parameter	Healthy dogs (100)**	OE dogs (100)	Total (200)
Age (1-10 years)	3.2 ± 2.7	3.4 ± 2.3	3.3 ± 2.5
Breed	Yorkshire Terrier (25)<br>Jack Russell Terrier (23)<br>German Shepherd (12)<br>Golden Retriever (14)<br>Labrador Retriever (10)<br>Border Collie (9)<br>Siberian Husky (7)	Yorkshire Terrier (22)<br>Jack Russell Terrier (18)<br>German Shepherd (14)<br>Golden Retriever (26)<br>Labrador Retriever (8)<br>English Cocker Spaniel (7)<br>Beagle (5)	Yorkshire Terrier (47)<br>Jack Russell Terrier (41)<br>German Shepherd (26)<br>Golden Retriever (40)<br>Labrador Retriever (18)<br>Border Collie (9)<br>Siberian Husky (7)<br>English Cocker Spaniel (7)<br>Beagle (5)
Gender (M/F)	53/47	57/43	110/90
Ear swab specimens (R/L)	51/49	47/53	98/102
Clinical signs*	-	Erythema (80)<br>Oedema (45)<br>Pruritus (87)<br>Otic discharge (72)	-

Data are presented descriptively as n(%) or mean ±SD. No statistical comparison was performed for demographic variables. ***:** Only observed in OE-affected dogs, ****:** Number of animals, **M/F:** Male/Female, **R/L:** Right/Left,
