# Supplementary material for: Culture and Metagenomic Insights into the Ear Microbiota in Dogs with Healthy Ears and Otitis Externa
Source: Vet Sci. 2026 Mar 6;13(3):250. doi: 10.3390/vetsci13030250 (PMC13030325; doi:10.3390/vetsci13030250)
Supplement: Supplementary file 1 [file vetsci-13-00250-s001.zip › Table S2.pdf]

Table S2. Frequencies of the bacteria defined from ear swab specimens

Bacteria		Dogs with healthy n=159	Species	Dogs with OE n=177
**Genus**	**Species**			
Staphylococcus		**65 (40.9)***		**77 (43.5)**
	S. pseudintermedius	39 (24.5)	*S. pseudintermedius*	54 (30.5)
	S. epidermidis	9 (5.7)	*S. epidermidis*	5 (2.8)
	S. aureus	4 (2.5)	*S. aureus*	3 (1.7)
	S. simulans	4 (2.5)	*S. schleiferi*	3 (1.7)
	S. hominis	2 (1.3)	*S. hominis*	2 (1.1)
	S. felis	2 (1.3)	*S. canis*	2 (1.1)
	S. canis	1 (0.6)	*S. felis*	2 (1.1)
	S. schleiferi	1 (0.6)	*S. simulans*	1 (0.6)
	S. auricularis	1 (0.6)	*S. delphini*	1 (0.6)
	S. capitis	1 (0.6)	*S. succinus*	1 (0.6)
	S. equorum	1 (0.6)	*S. xylosus*	1 (0.6)
			S. warneri	1 (0.6)
			S. condimenti	1 (0.6)
Bacillus		**30 (18.9)**		**30 (16.9)**
	B. cereus	6 (3.8)	*B. cereus*	7 (4)
	B. pumilus	6 (3.8)	*B. pumilus*	5 (2.8)
	B. megaterium	3 (1.9)	*B. licheniformis*	3 (1.7)
	B. simplex	3 (1.9)	*B. paralicheniformis*	2 (1.1)
	B. amyloliquefaciens	3 (1.9)	*B. weihenstephanensis*	1 (0.6)
	B. altitudinis	2 (1.3)	*B. oleronius*	1 (0.6)
	B. subtilis	1 (0.6)	*B. oceanisediminis*	1 (0.6)
	B. thuringiensis	1 (0.6)	*B. stratosphericus*	1 (0.6)
	B. licheniformis	1 (0.6)	*B. endophyticus*	1 (0.6)
	B. safensis	1 (0.6)	*B. safensis*	1 (0.6)
	Bacillus spp.	3 (1.9)	*B. atrophaeus*	1 (0.6)
			B. velezensis	1 (0.6)
			Bacillus spp.	5 (2.8)
Enterococcus		**13 (8.2)**		**14 (7.9)**
	E. faecalis	5 (3.1)	*E. faecalis*	5 (2.8)
	E. faecium	5 (3.1)	*E. faecium*	5 (2.8)
	E. hirae	2 (1.3)	*E. hirae*	2 (1.1)
	E. casseliflavus	1 (0.6)	*E. durans*	1 (0.6)
			E. cecorum	1 (0.6)
Clostridium	***C. perfringens***	**6 (3.8)**	***C. perfringens***	**8 (4.5)**
Escherichia	***E. coli***	**6 (3.8)**		**6 (3.4)**
			E. coli	5 (2.8)
			E. hermannii	1 (0.6)
Macrococcus		**6 (3.8)**	***M. caseolyticus***	**1 (0.6)**
	M. caseolyticus	5 (3.1)		
	M. canis	1 (0.6)		
Streptococcus		**5 (3.1)**	***S. canis***	**5 (2.8)**
	S. lutetiensis	2 (1.3)		
	S. uberis	1 (0.6)		
	S. suis	1 (0.6)		
	S. minor	1 (0.6)		
Paenibacillus		**3 (1.9)**		**6 (3.4)**
	P. odorifer	1 (0.6)	*P. vulneris*	2 (1.1)
	P. polymyxa	1 (0.6)	*P. illinoisensis*	1 (0.6)
	Paenibacillus sp.	1 (0.6)	*P. phoenicis*	1 (0.6)
			P. macerans	1 (0.6)
			Paenibacillus sp.	1 (0.6)
Corynebacterium		**3 (1.9)**		**3 (1.7)**

	C. freneyi	2 (1.3)	*C. auriscanis*	1 (0.6)
	C. ureicelerivorans	1 (0.6)	*C. amycolatum*	1 (0.6)
			Corynebacterium sp.	1 (0.6)
Pseudomonas				**5 (2.8)**
			P. aeruginosa	4 (2.3)
			P. stutzeri	1 (0.6)
Proteus	***P. mirabilis***	**2 (1.3)**	***P. mirabilis***	**4 (2.3)**
Aneurinibacillus				**2 (1.1)**
			A. aneurinilyticus	1 (0.6)
			A. migulanus	1 (0.6)
Micrococcus	*M. luteus*	3 (1.9)	*M. luteus*	1 (0.6)
Enterobacter	*E. cloacae*	3 (1.9)	*E. cloacae*	1 (0.6)
Klebsiella			*K. pneumoniae*	3 (1.7)
Aerococcus	*A. urinaeequi*	1 (0.6)	*A. viridans*	1 (0.6)
Vagococcus	*V. fluvialis*	1 (0.6)		
Kocuria	*K. rhizophila*	1 (0.6)		
Jeotgalicoccus			*J. halotolerans*	1 (0.6)
Gemella			*G. sanguinis*	1 (0.6)
Weissella			*W. cibaria*	1 (0.6)
Faecalicoccus			*Faecalicoccus* sp.	1 (0.6)
Brevibacillus			*B. borstelensis*	1 (0.6)
Calidifontibacillus	*C. erzurumensis*	1 (0.6)		
Ligilactobacillus	*L. murinus*	1 (0.6)		
Lysinibacillus	*L. fusiformis*	1 (0.6)		
Peribacillus	*P. simplex*	1 (0.6)		
Psychrobacillus	*P. mangrovi*	1 (0.6)		
Eubacterium	*E. tenue*	1 (0.6)		
Streptomyces	*Streptomyces* sp.	1 (0.6)		
Acinetobacter	*A. lwoffii*	1 (0.6)		
Pasteurella			*P. multocida*	1 (0.6)
Moraxella	*M. canis*	1 (0.6)	*M. osloensis*	1 (0.6)
Neisseria	*N. flavescens*	1 (0.6)	*N. flavescens*	1 (0.6)
Prolinoborus	*P. fasciculus*	1 (0.6)		
Pantoea			*Pantoea agglomerans*	1 (0.6)
Massilia			*Massilia varians*	1 (0.6)

***:** Number of the isolates (%), **n:** Total number of isolates defined in the ears of healthy and otitis externa dogs.
