# Supplementary figures and images for: Culture and Metagenomic Insights into the Ear Microbiota in Dogs with Healthy Ears and Otitis Externa
Source: Vet Sci. 2026 Mar 6;13(3):250. doi: 10.3390/vetsci13030250 (PMC13030325; doi:10.3390/vetsci13030250)

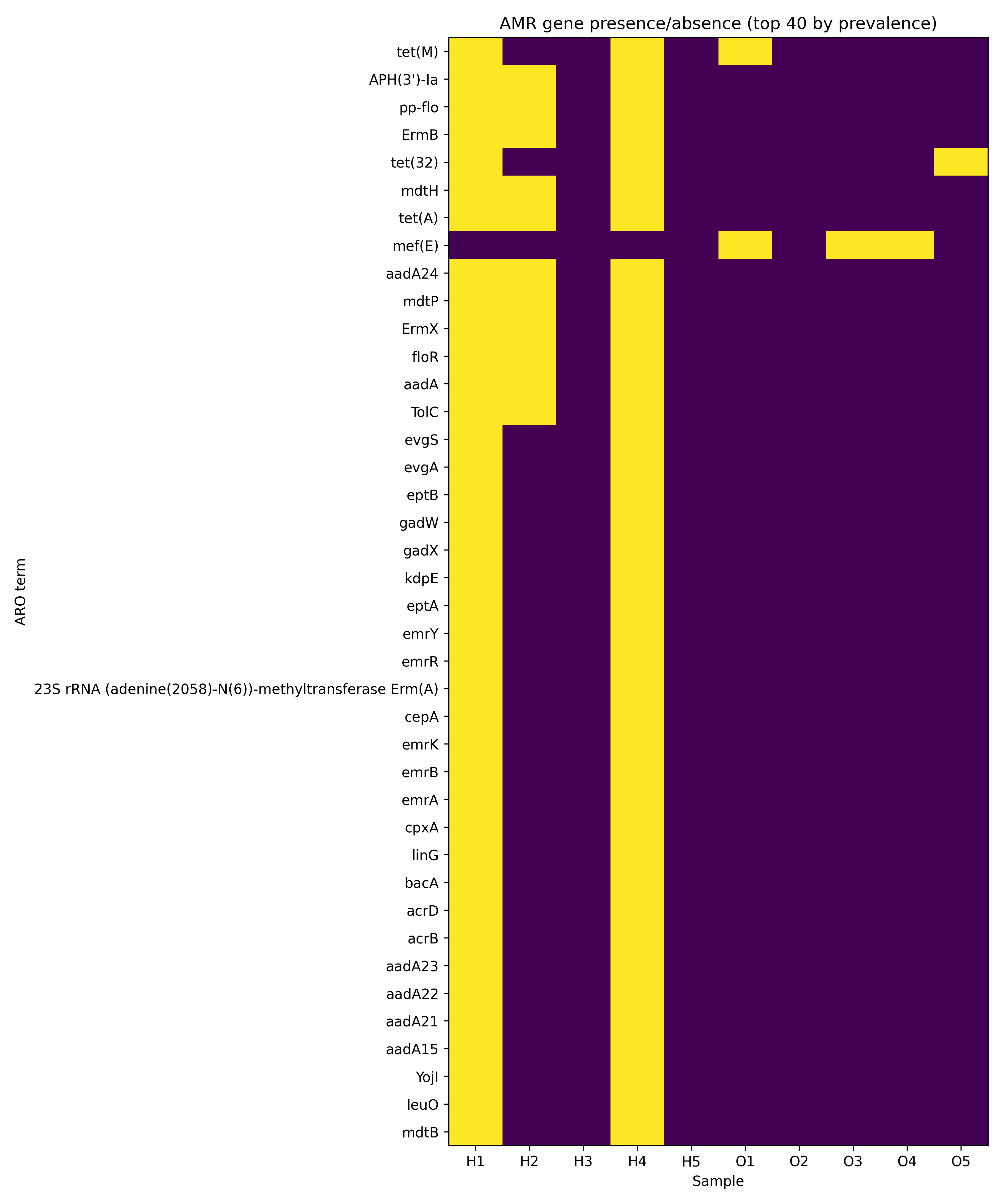

Supplement: Supplementary file 1 [file vetsci-13-00250-s001.zip › Figure S1.png]

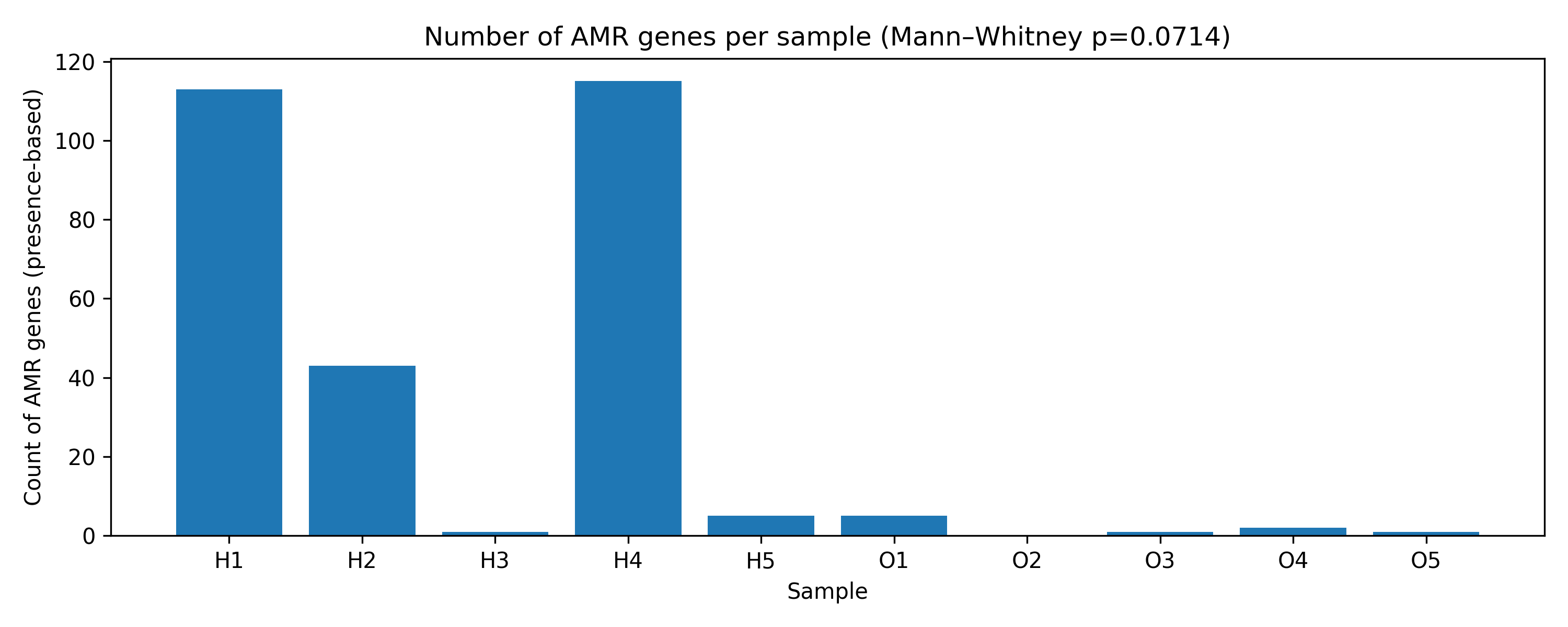

Supplement: Supplementary file 1 [file vetsci-13-00250-s001.zip › Figure S2.png]

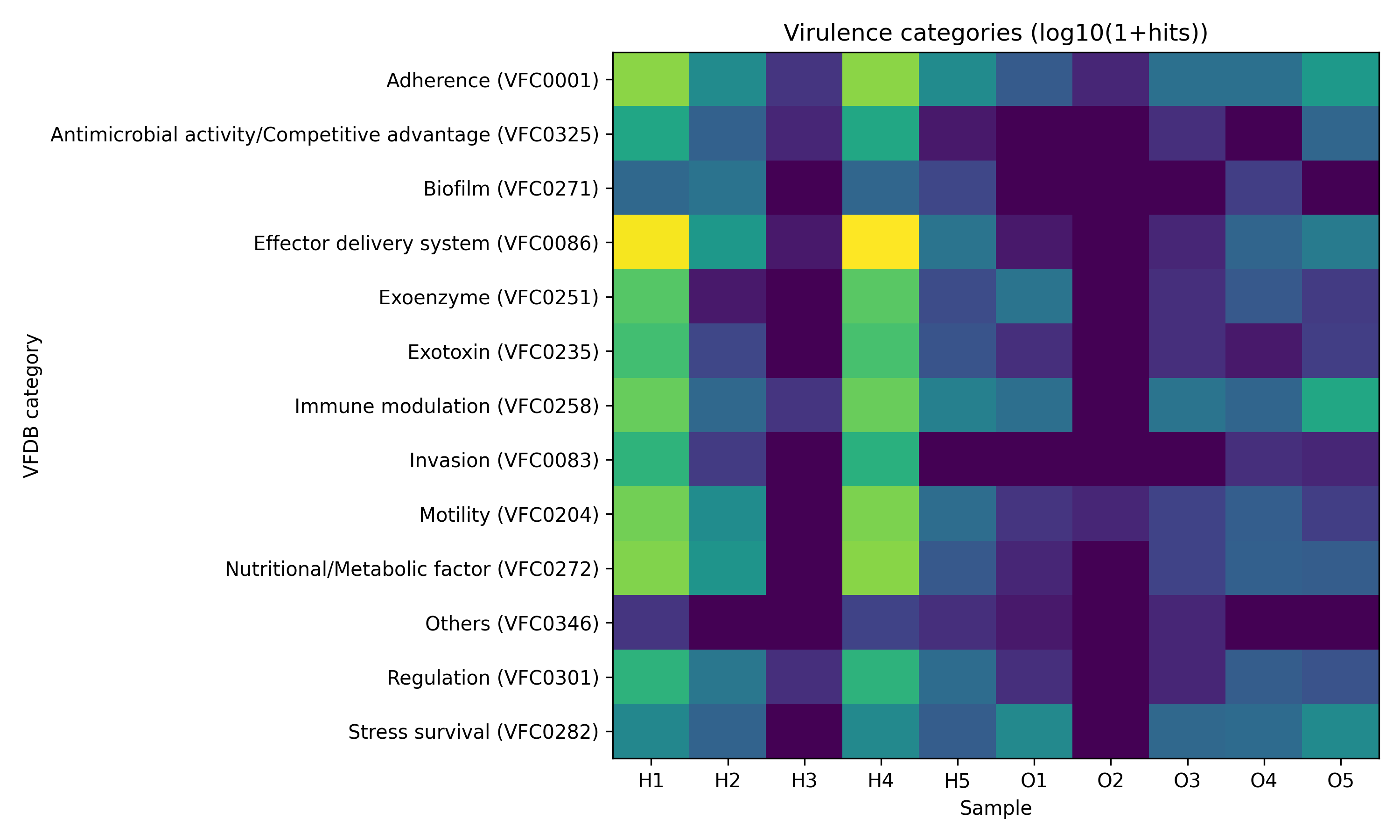

Supplement: Supplementary file 1 [file vetsci-13-00250-s001.zip › Figure S3.png]

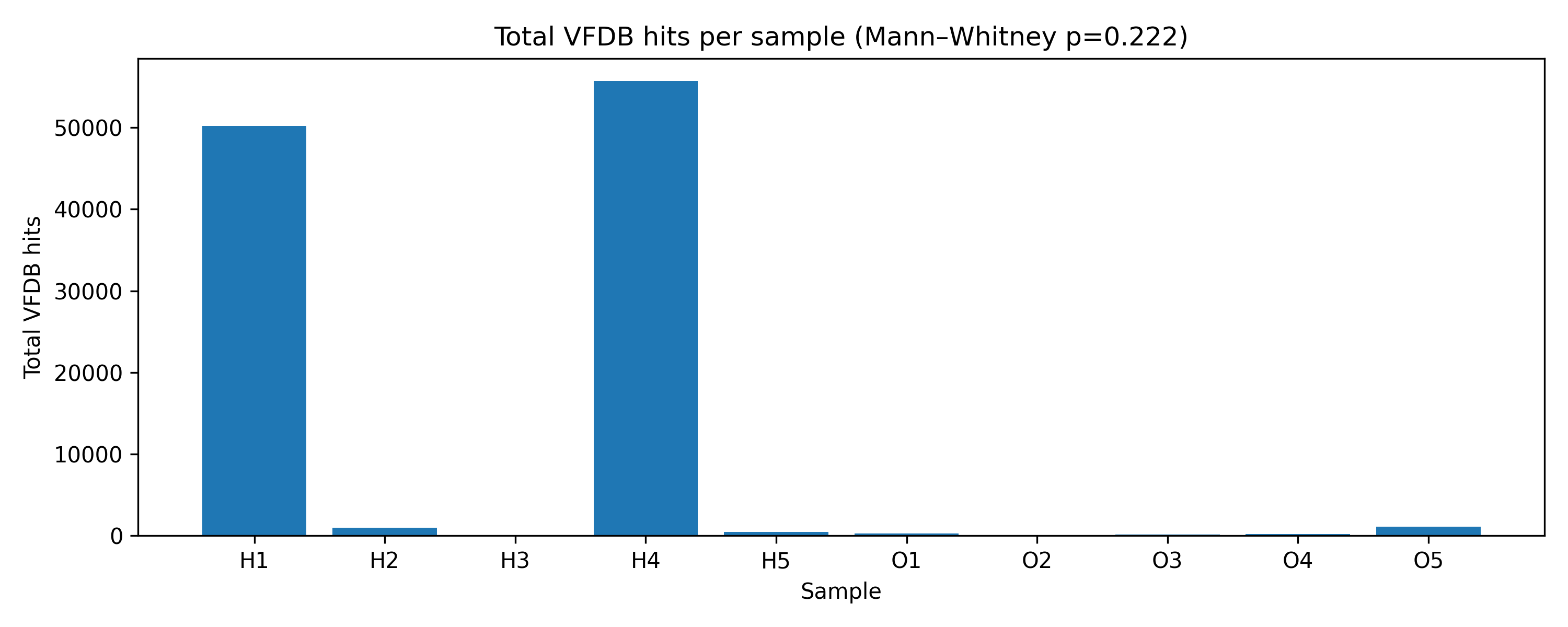

Supplement: Supplementary file 1 [file vetsci-13-00250-s001.zip › Figure S4.png]
